# Using Social Media for Qualitative Health Research in Danish Women of Reproductive Age: Online Focus Group Study on Facebook

**DOI:** 10.2196/24108

**Published:** 2021-05-31

**Authors:** Camilla Gry Temmesen, Henriette Svarre Nielsen, Heidi Lene Myglegård Andersen, Kathrine Birch Petersen, Jane Clemensen

**Affiliations:** 1 HCA Research, Hans Christian Andersen Children’s Hospital Odense University Hospital Odense Denmark; 2 Centre for Innovative Medical Technology Odense University Hospital Odense Denmark; 3 Department of Nursing University College Absalon Roskilde Denmark; 4 Institute of Clinical Research, Faculty of Health Sciences University of Southern Denmark Odense Denmark; 5 Department of Obstetrics and Gynecology Copenhagen University Hospital Hvidovre Denmark; 6 Department of Clinical Medicine University of Copenhagen Copenhagen Denmark; 7 TFP Stork Fertility The Fertility Partnership DK Copenhagen Denmark

**Keywords:** internet, social media, Facebook, online focus groups, women, reproduction, reproductive age, motherhood, participatory design

## Abstract

**Background:**

Social media platforms provide new possibilities within health research. With Facebook being the largest social network in the world, it constitutes a potential platform for recruitment and data collection from women of reproductive age. Women in Denmark and in other Western countries postpone motherhood and risk infertility due to their advanced age when they try to conceive. To date, no study has explored Danish women’s reflections on the timing of motherhood within a social media setting.

**Objective:**

The aim of this study was to explore the challenges and opportunities of using Facebook as a platform for qualitative health research in Danish women of reproductive age.

**Methods:**

This study was a qualitative study based on 3 online focus groups on Facebook with 26 Danish women of reproductive age discussing the timing of motherhood in January 2020.

**Results:**

Conducting online focus groups on Facebook was successful in this study as the web-based approach was found suitable for developing qualitative data with women of reproductive age and made recruitment easy and free of charge. All participants found participating in an online focus group to be a positive experience. More than half of the women participating in the online focus groups found it advantageous to meet on Facebook instead of meeting face-to-face.

**Conclusions:**

Conducting online focus groups on Facebook is a suitable method to access qualitative data from women of reproductive age. Participants were positive toward being a part of an online focus group. Online focus groups on social media have the potential to give women of reproductive age a voice in the debate of motherhood.

## Introduction

### Background of This Study

Within the last decade, social media platforms have gained a prominent role in the way people communicate and interact with one another and have enabled people to be connected and accessible to a greater extent. Social media offer multiple possibilities such as chat forums, blogs, virtual worlds, and social networks such as Facebook, Twitter, Instagram, and SnapChat. Facebook is currently the largest and most popular social network in the world with an average of 1.73 billion daily active users and an average of 2.6 billion monthly active users worldwide, as reported in 2020 [1]. In Denmark too, Facebook constitutes the most popular social media with 77% of all Danes having a Facebook profile [2]. In 2018, 95.3% of Danes aged 19-34 years had a Facebook profile followed by 86% of Danes aged 35-54 years [2], with 72% using Facebook daily [3]. Social media networks are popular among women of reproductive age. On Facebook, women can engage in different web-based groups, such as pregnancy, due date, and baby groups with the possibility of sharing experiences, photos, life stories, and to be a part of a virtual community with other future parents. However, web-based groups for women who have not yet had children are not common neither are the possibilities for discussing the future with or without children online with other women of reproductive age. Within health care, social media has increasingly become a way of informing, communicating, interacting, and engaging with people. Previous studies have explored the use of social media within health research, for example, a study that explores mommy bloggers disseminating breast cancer risk information [4], a study that uses Facebook to deliver smoking cessation treatment to young adults [5], or a study using online support groups for women with endometriosis [6].

### Web-Based Recruitment and Web-Based Data Collection

Within qualitative research, focus groups are a popular data collection method. However, this method is often associated with challenges to recruit enough eligible participants, and inclusion of participants across the country can be time-consuming and economically and practically challenging [7]. Web-based recruitment is a well-known strategy in health research, especially pointed at “hard-to-reach” populations [8] such as marginalized groups or people who do not have access to the internet. Choosing traditional recruitment approaches such as posters, brochures, or personal approaching in health clinics when recruiting women of reproductive age who have not yet had children can be challenging, as most of these women do not have the need for consulting health professionals or have a natural web-based community. When planning this study, Facebook seemed to have the potential for recruitment and collection of data, since Danish women in the age group of 18-45 years constitute the most active users on Facebook with 86.5% using Facebook several times a day or almost every day compared to 77.9% of men of the same age group (Index Danmark, Gallup, Social media: use of Facebook by age and sex, unpublished data, 2019). Women tend to engage on Facebook by liking and sharing content [9], which can contribute to a faster recruitment process. Online focus groups, also referred to as virtual focus groups, are known from social science research, but online focus groups have become an interesting methodological approach within health science too. Williams et al [10] defines online focus groups as *“A selected group of individuals who have volunteered to participate in a moderated, structured online discussion in order to explore a particular topic for the purpose of research.”* Previously, online focus groups have been used in a study exploring DNA paternity testing with single mothers with young babies, for whom it was difficult to participate in face-to-face focus groups [11,12] and in another study as an approach to explore breastfeeding women’s use of social media [13].

### Timing of Motherhood

Danish women’s age at the birth of their first child has risen significantly throughout the last 50 years. In 1969, Danish women were 23.3 years old when giving birth to their first child, which has risen to 29.5 years of age in 2019 [14]. Postponing motherhood increases the risk of infertility and pregnancy complications such as miscarriage [15,16], chromosomal abnormalities [15,17], hypertensive complications, fetal growth restrictions, fetal death [18], and birth complications such as cesarean section [19]. The higher the age also means an increased risk of other risk factors, including overweight, sexually transmitted diseases, cancer, endometriosis, or environmental exposures, for example, hormone disruptive chemicals that may affect female fertility [15]. Postponement of motherhood is not unique to Denmark but has been reported in other Western and Northern European countries too [20-24].

### Aim and Purpose of This Study

This paper presents an exploration of a qualitative methodological approach using Facebook to collect data from women of reproductive age, discussing their thoughts on the timing of motherhood. The aim of this study was to explore Facebook as a platform for qualitative health research within the reproductive field by recruiting women who had not yet had children into an online focus group discussing the timing of motherhood as a way to explore the challenges and opportunities of using Facebook to collect qualitative data. The online focus group was a part of a Participatory Design research-inspired study, which included a literature study and individual interviews with women of reproductive age. To the best of our knowledge, no previous research has addressed the approach of conducting online focus groups with a group of women of reproductive age in a social media setting. This paper focuses solely upon the methodological approaches of conducting online focus groups. In-depth empirical data from the online focus groups will be presented elsewhere.

## Methods

### Study Design

This study was conducted as an asynchronous text-based online focus group study on Facebook. In previous literature, focus groups within computer-mediated settings were also known as virtual focus groups. In this study, we prefer using the term “online focus groups” describing focus group activities connected to the internet [25], as we see the term “virtual” referring to a 3D image or environment that can be interacted with in a seemingly real or physical way by a person using special electronic equipment, as known from virtual reality [26]. Facebook was chosen as a web-based platform for the online focus groups because of its popularity, user friendliness, and the possibility to create private online groups. Prior to this study, a pilot study in 2016 with 14 Danish women tested Facebook as a platform for discussing the timing of motherhood in a social media setting, and Facebook was then found to be a suitable platform for this target group [27].

### Participant Recruitment

We used a purposive recruitment strategy where participants were invited to the online focus groups if they met the following inclusion criteria: women of reproductive age defined as 18-45 years, with no children, regardless of whether they want to have children in the future, single or in a relationship, capable of speaking and writing Danish, and having a personal Facebook profile. Participants were recruited using the snowball sampling method on Facebook in the beginning of January 2020, initially through a recruitment post shared within the main author’s social network. The recruitment post, containing information about the project, was shared 287 times, reaching potential participants in all 5 regions of Denmark. None of the participants knew the facilitator prior to participation but were recruited through mutual connections within the social network. Participants who fulfilled the inclusion criteria contacted the facilitator (main author) by mail or *Messenger* (Textbox 1) [28] and answered a short web-based demographic questionnaire with a written consent to participate. We aimed to have 8-10 participants for each group, which was achieved for 2 out of 3 groups. Recruitment lasted for 20 days and finished when the desired number of participants were reached. Two potential participants withdrew from the study without explanation prior to data collection.

**Social media dictionary.**

*Post:* A piece of writing, image, or other item of content published online, typically on a blog or social media website.

*Tagging:* Linking a person to a comment or a post by adding his/her profile name.

*Notifications:* A message, email, icon or another symbol that appears when an app wants you to pay attention.

*Emoji:* A small digital image or icon used to express emotions.

*Reaction symbols:* A series of 6 animated “emoji” reactions that customers can add when responding to a post. Reaction symbols are Facebook’s way of facilitating an emotional conversation online.

*Messenger:* Messenger is a free mobile messaging app developed by Facebook, used for instant messaging, sharing photos, videos, audio recordings, and group chats.

### Participant Characteristics

A total of 26 women agreed to participate in the online focus groups lasting for 4 days (January 27-30, 2020). Participants were sent a personal invite linked to their private Facebook profiles to join a private Facebook group that was only visible to the facilitator and the participants. Participants were divided into 3 age groups with a private Facebook group created for each group. The participants were divided into different age groups with the intention to obtain insight into the potential similarities or differences among (1) younger women aged 18-24 years (n=8), (2) women aged 25-34 years (n=11), who according to their age are most likely to consider having children, and (3) women aged 35-45 years (n=7), who represent being at advanced maternal age defined as 35 years and older.

### Online Focus Groups

The online focus groups were initiated by an elaboration of how the focus groups would proceed. As recommended by Abrams and Gaiser [29], participants were encouraged to make a short presentation of themselves as a way of enabling trust and making them feel comfortable. Since the participants used their personal Facebook profile, their identities were inevitably visible to other participants in the online focus groups, which is why we encouraged confidentiality between the participants. Participants were briefed that they could expect a daily topic to be uploaded and discussed within the group and that the estimated time each participant was expected to spend in the group was approximately 15 minutes per day. An interview guide inspired by traditional focus groups was prepared, as recommended by Malterud [30] and Skelton et al [13] in their study of breastfeeding mothers’ use of social media. The interview guide focused on 4 broad main themes: (1) *considerations regarding when to have children,* (2) *the influence of others,* (3) *age and women's fertility,* and (4) *fertility knowledge.* Each theme represented a daily topic and was visually presented with a banner (photo or illustration) to make it easier to navigate between the various discussion threads in the group. The facilitator uploaded a new topic with support questions to be discussed each of the 4 days the online focus groups lasted, which addressed the 4 main themes of the interview guide. A range of open-ended support questions was designed to facilitate reflections on the topic and to promote a dialogue between the participants (Textbox 2).

**An example of a topic for discussion in the online focus groups on the second day.**

Today I would ask you to discuss the following issue: Do you feel that the outside world influences your thoughts about having children?

Supporting questions:

- Feel free to define what you mean by the outside world (is it your possible partner, family, parents, siblings, grandparents), friends/girlfriends, politicians, media, community or otherwise?)
- Do you find that the outside world positively affects you to have children?
- Do you find that the outside world negatively affects you to have children?
- Does that make you want to have children earlier or later?
- Have you told your outside world about your considerations or choices regarding your thoughts on having children?
- Who are your loved ones that you talk to about your thoughts on having children? (eg, partner, parent, sibling, friends/girlfriends, healthcare professionals or others?)
- Is it hard to talk to anyone else about these thoughts? Why/why not?

**Figure 1:**
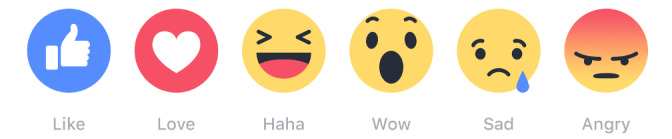
Facebook reaction symbols.

Apart from text-written communication, participants had the possibility of using Facebook's *reaction symbols* [31] (Figure 1) [32] to acknowledge comments or posts from coparticipants. Reaction symbols are Facebook’s way of facilitating an emotional conversation online [31,33]. The online focus groups were asynchronous, meaning that participants could access the online focus groups at a time that suited them best during the day rather than real-time online focus groups where participants access the online focus groups at a predefined time [34]. Three days after the online focus groups were completed, participants received a web-based debriefing questionnaire to anonymously evaluate how they experienced participating in an online focus group. A debriefing can give the researcher insight into group dynamics and how the participants experienced being a part of the focus group [35,36]. A total of 464 comments or 97 full pages of transcripts were generated from the 3 online focus groups and were copied as written by the participants into a text-based document, including other forms of *interactions,* for example, uploaded photos or videos, *emojis* [37], and punctuation. Identifiable profile names were replaced with pseudonyms and profile pictures were excluded from the analysis. As the native language of the participants was Danish, data were translated to English after analysis to reduce the risk of important pointers being lost in translation.

### Ethical Considerations

This study was notified to the Regional Ethical Committee as well as the Danish Data Protection Agency, but in Denmark, interview studies no longer require approval [38]. This study followed the principles of the Helsinki Declaration [39] and The Danish Code of Conduct for Research Integrity [40], in which participants received written information with the purpose of the study, just as they were informed about the confidentiality and the right to withdraw from the study. As Facebook users, all participants had accepted the Facebook Terms of Service and Facebook Data Policy prior to participation in this study but were reminded of these policies when invited to the study. The online focus groups were created as private Facebook groups, meaning that all content within the groups was only visible to the facilitator and the participants. Only the facilitator could invite participants to the group. All groups were deleted from Facebook at 1 month after participation.

## Results

### Participant Characteristics

The age range of the participants was 18-43 years, which was considered as a representative age span for women of reproductive age. Approximately 35% (9/26) of the women were single and 65% (17/26) were in a relationship, of which 9% (2/26) were married, 39% (10/26) were in a relationship and cohabiting, and 19% (5/26) were in a relationship but not cohabiting. All 5 regions of Denmark were represented with 58% (15/26) of the women living in urban areas and 42% (11/26) living in provincial areas. The majority of the women (21/26, 81%) had a high educational level with 42% (11/26) having a medium higher education or 39% (10/26) having a higher education. One participant discovered she was pregnant during the data collection but was not excluded from the group since she was at a very early stage of pregnancy. These demographical data are shown in Table 1.

**Table 1:** Demographics of the women of reproductive age included in the online focus groups in this study.

Participant ID	Pseudonym	Age (years)	Region of living	Area of living	Educational level	Relationship status
W1	Anna	39	Southern Denmark	Rural	Medium higher education (3-4.5 years)	Single
W2	Amelia	31	Southern Denmark	Urban	Medium higher education (3-4.5 years)	Single
W3	Ava	31	Zealand	Rural	Medium higher education (3-4.5 years)	Single
W4	Beatrice	32	Capital	Urban	Higher education (5-6 years)	Single
W5	Catherine	30	Northern Denmark	Rural	Higher education (5-6 years)	In a relationship, cohabiting
W6	Charlotte	28	Capital	Rural	Higher education (5-6 years)	In a relationship, not cohabiting
W7	Emily	43	Capital	Urban	Medium higher education (3-4.5 years)	Single
W8	Hannah	32	Capital	Urban	Medium higher education (3-4.5 years)	In a relationship, cohabiting
W9	Helen	35	Zealand	Rural	Higher education (5-6 years)	In a relationship, not cohabiting
W10	Julia	23	Capital	Urban	Medium higher education (3-4.5 years)	In a relationship, not cohabiting
W11	Josephine	32	Capital	Urban	Medium higher education (3-4.5 years)	In a relationship, not cohabiting
W12	Lauren	38	Central Denmark	Urban	Higher education (5-6 years)	Single
W13	Lily	27	Capital	Urban	Higher education (5-6 years)	In a relationship, cohabiting
W14	Megan	23	Zealand	Rural	Medium higher education (3-4.5 years)	In a relationship, cohabiting
W15	Mia	25	Zealand	Rural	Medium higher education (3-4.5 years)	In a relationship, cohabiting
W16	Olivia	35	Capital	Rural	Shorter higher education (2-3 years)	In a relationship, cohabiting
W17	Sarah	25	Central Denmark	Urban	Other	Single
W18	Sophie	33	Southern Denmark	Rural	Higher education (5-6 years)	In a relationship, cohabiting
W19	Susan	19	Zealand	Rural	Primary school	In a relationship, cohabiting
W20	Tessa	29	Southern Denmark	Urban	Medium higher education (3-4.5 years)	Single
W21	Tori	36	Southern Denmark	Urban	Higher education (5-6 years)	Single
W22	Tracy	38	Southern Denmark	Urban	Higher education (5-6 years)	In a relationship, cohabiting
W23	Vanessa	26	Capital	Urban	Higher education (5-6 years)	Married
W24	Vera	18	Southern Denmark	Urban	High school	In a relationship, not cohabiting
W25	Veronica	24	Capital	Urban	Medium higher education (3-4.5 years)	Married
W26	Victoria	20	Capital	Rural	High school	In a relationship, cohabiting

### Participant Interactions

Facebook provided a *notification* every time a new comment or a reaction was added in the group, making it possible for the participants and the facilitator to keep track on the activity in the groups. Where the majority of the participants appeared to be the most active within the groups during day and evening time, often being active online at the same time, which at times made the online focus groups synchronous, a few participants were more likely to contribute with their comments late at night. All posts uploaded by the facilitator were seen by all the participants, but not every participant contributed to the discussion. Facebook offers a feature that indicates whether the participants have seen the posts or not, which make it visible to the facilitator if some participants were not active in the group. Participants were considered as low responders if they contributed less than 5 comments or interactions (n=3), medium responders if they contributed 5-15 comments or interactions (n=15), and as high responders if they contributed 15 comments or interactions and more (n=8) (Table 2). The number of comments ranged from 2 comments or interactions from 1 participant to 45 comments or interactions from another participant. None of the participants left the online groups or withdrew their wish to participate in the study during data collection. Overall, there was a high engagement level in all 3 online focus groups fostering rich discussions on the topics with an average of 142 comments per group (Table 2). Participants naturally applied the different emojis and reaction symbols as a way of interacting nonverbally with each other (Textbox 3).

**Table 2:** Interaction among the women of reproductive age in online focus groups categorized by age and type of responder.

Responses	18-25 years age group (n=8)	26-34 years age group (n=11)	35-45 years age group (n=7)
Low responders^a^ (n=3)	1	1	1
Medium responders^b^ (n=15)	7	5	3
High responders^c^ (n=8)	0	5	3
Comments per group	75	195	157
Comments per person (median)	6.5	15	15

^a^<5 comments.

^b^5-15 comments.

^c^>15 comments.

In all groups, there was a friendly informal tone among the participants. Between the age groups, there were some differences in how often and in which way participants interacted with each other. While participants in age group 26-34 years and age group 35-45 years often addressed specific questions or comments to other participants by *tagging* [41] (Textbox 3), participants in the age group of 18-25 years primarily answered the questions from their own point of view and did not interact as much with each other as the other groups did, which led to markedly fewer comments and less lively discussions in this age group. Two participants did not contribute to the discussions; they only contributed with a presentation of themselves, thereby being merely passive participants.

**Examples of text interactions among Tori, Anna, and Lauren (age group 35-45 years) in the online focus group on Facebook.**

Tori (W21): Anna, *I always say that there will be some divorced men with cute children, where you can become a bonus mother when the marital crises hit 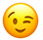 . Then you can experience couple relationships and the mother role too, if you have the needs. Then they never quite know what to answer and the conversation changes topic 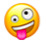
*

Anna (W1): Tori, * hey good tactics! I like it 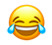
*

Lauren (W12): *Yes, I have also met people who say, “you will regret” or “in 5 years we’ll see you with a baby on the arm”. Very condescending, I think...*

### Facilitator Role

The facilitator applied a low management role to the discussions, which, apart from addressing the daily topic and supporting questions consisted of having a more observant role in the discussions. However, the facilitator ongoingly addressed specific questions either as general questions within the groups if the interview topics did not appear naturally within the discussions among the participants or asked individual questions to get a participant to elaborate on her answer (Textbox 4).

**An example of a communication thread between the facilitator and a participant.**

Susan (W19): *(…) I think everyone has an opinion on when one should or should not have children. Whether it is one or the other they think, then it is completely natural for me to listen to the arguments they come up with. I personally want to be a young mother, and there are many who have an attitude to it. And I can be negatively affected in relation to my attitude to have children at a young age, as there are so many who do not think it is a good idea - and since it is one's primary circle, it's hard not to be influenced by their opinion (…)*

Facilitator: Susan,* can you say more about how you experience the negative attitude towards your desire to become a mother at a young age?*

Susan (W19): Facilitator, *I often think that people rule out the possibility that you can have experiences after having children (…)*

### Responses in the Debriefing Questionnaire

Out of 26 women, 89% (23/26) answered the debriefing questionnaire. When asked why they decided to participate in the study, 65% (15/23) of the women stated that they felt obligated to participate in the research aiming at them, 61% (14/23) of the women participated because they found it exciting to be a part of an online focus group, and 48% (11/23) of the women participated in the study because the topic motherhood was found to be interesting (multiple answers were possible). When asked on a 5-point Likert Scale (very high degree, some degree, low degree, very low degree, don’t know), all participants agreed that participating in the online focus groups was a positive experience, with 70% (16/23) agreeing to a high degree and 30% (7/23) agreeing to some degree. Overall, 61% (14/23) of the women felt that participating in an online focus group made them feel as a part of a community with 4% (1/23) who felt to a high degree and 57% (13/23) who felt to some degree that they were a part of a community, whereas 26% (6/23) felt to a low degree and 4% (1/23) felt to a very low degree that they were a part of a community. Approximately 17% (4/23) of the participants felt to a high degree and 43% (10/23) felt to some degree that it was an advantage to meet on Facebook instead of meeting face-to-face, whereas 13% (3/23) of the participants felt to a low degree and 9% (2/23) of the participants felt to a very low degree that it was advantageous to meet on Facebook. One participant stated that “I think that it would have supported the internal dialogue between participants having been physically together, since communication is so much more than words (…) However, I probably wouldn’t have had the opportunity to attend if it required physical attendance, so I think this is a great solution!” The majority of the women spent more time than the estimated 15 minutes per day participating in online focus groups, with 35% (8/23) of the participants spending 5-15 minutes daily, 39% (9/23) of the participants spending more than 15 minutes, and 26% (6/23) of the participants spending more than 30 minutes daily. One participant claimed that “It was a little more time-consuming than I expected, and because I was lacking time, I attended less than I had hoped for.”

## Discussion

### Overview of This Study

The aim of this study was to explore the challenges and opportunities of using Facebook as a platform for qualitative health research in women of reproductive age*.* This study represents 26 Danish women from 3 different age groups reflecting their thoughts upon the timing of motherhood. Rather than aiming to conduct more online focus groups, we decided to include participants who were different in terms of age, relationship status, and area of living (urban or rural), and from the 3 online focus groups, we obtained rich data on reflections on the timing of motherhood.

### Facebook as a Platform

Using Facebook as a platform for conducting online focus groups with women of reproductive age was beneficial for this study, as it made recruitment and data collection free of charge and inclusion of women across the country without the need for covering travelling and venue expenses. Another benefit was that through Facebook, we gained access to participants and data by creating a temporary online space for women of reproductive age, which could otherwise have been difficult, as this group of women do not have a natural web-based meeting space and can be hard to reach using traditional recruitment methods. It was an advantage that the recruitment as well as the data collection took place on the same platform, since Facebook has user-friendly features and was well-known to the participants. When conducting online focus groups with women of reproductive age on Facebook, we met participants on a media platform that they were already familiar with [42], which makes recruitment and adaptation to the research tool easier. Transcriptions were easy, as participants generated the transcripts themselves and the text-based data were copy-pasted for analysis, which increases the accuracy of the transcripts and removes the potential for error [10,43]. However, the anonymization of the participants and the use of emojis, reaction symbols, punctuation, photos, and videos uploaded by participants required some preparation of data prior to analysis.

### Asynchronous Versus Real-Time Online Focus Groups

The asynchronous interaction in the online focus groups meant that participants had the freedom to participate at a time that suited them best, thereby enhancing flexibility and convenience. Another advantage was that participants had the ability to return to a previously asked question and elaborate on their answers or contribute with their points of view later on if they did not have the opportunity to answer immediately. This gave participants more time for reflection before submitting a response, thereby enabling richer collection of data. This finding is supported by Lijadi and van Schalkwyk [34] in their study of Third Culture Kids. A disadvantage of asynchronous online focus groups was that when a topic was being discussed, participants had to wait for a response from the other participants before the discussion could start properly. Compared to real-time chat-based interviews or traditional focus groups, this asynchronous approach may cause participants not to respond as spontaneously because they consider their answers [36]. The asynchronous approach required that participants checked updates in the Facebook group several times a day to keep up with the discussions, with the risk of overlooking some comments. The danger of Facebook being a platform that can be accessed anytime and anywhere is that participants can easily be distracted and forget to return to a discussion thread. However, Facebook has a helpful tool in the form of *notifications* [44] that reminded participants about new comments or posts in the group. Some participants were online simultaneously, which from time to time unintendingly caused the online focus groups to have the character of being synchronous, because participants responded and interacted in real time. Being online at the same time made the discussions lively and caused participants to interact more. However, in-depth comments often required participants to reflect upon their answers, which made the asynchronous approach more suitable, but a combination of both synchronous and asynchronous interactions seems to be ideal. This finding is supported by Graffigna and Bosio [45] in their study of young people and HIV/AIDS, who state that a combination of synchronous and asynchronous approaches to online-focused discussions maximizes the richness of data collected by fostering both immediate interaction and considered responses. However, Bloor et al [46] state that when doing synchronous online focus groups, there is a risk of the discussions going too fast, which requires greater attention from participants and the facilitator, when multiple discussion threads need to be controlled simultaneously. One participant elaborated on participating in an online focus group on Facebook compared to a face-to-face focus group and stated that she would probably not have participated if she were to meet physically. This illustrates that some participants who would otherwise have declined to participate in a face-to-face focus group owing to time restraints agree to participate when the research takes place online. Lijadi and van Schalkwyk [34] suggested that online focus group participants tend to contribute with shorter comments than participants in face-to-face focus groups. This contradicts with our findings since the majority of the comments in this study tended to be relatively long and reflective. In our experience, being a part of an online focus group makes it possible for participants to reflect upon thoughts and elaborate on them, which in our study contributed to richness in data. Williams et al [10] argue that written language can enhance the communication for people who feel more comfortable in expressing their feelings in text with the ability to express rich feelings and detailed reflections rather than expressing themselves face-to-face. In their study of online focus group discussions within the setting of pediatric oncology, Tates et al [43] argued that owing to the nature of being online without the physical presence, online focus groups can increase self-disclosure and lead to a higher level of interaction among participants.

### Facilitator Role

When doing research on social media, as a researcher, you need to adapt to the platform being used by being familiar with the formal codes of conduct (in this case the Facebook Policy) and the informal codes of conduct (netiquette, ie, a set of rules for acceptable online behavior) and know the jargons of the web-based platform to help participants navigate on the platform and to appear as a natural member of the group. Halkier [36] emphasizes that when planning online focus groups, it is important to consider the level of management needed. Online discussions can easily get off-track, which is why we considered it necessary to define a daily topic for participants to discuss, thereby incorporating it in the interview guide, supported by a number of support questions and associated banners to visually support the topic. Having too strictly formulated questions can cause participants to only respond to what they are asked, which will cause the discussions to be less nuanced [36]. In this study, this did not seem to be the case, because when participants occasionally moved away from the original question, new and interesting angles emerged, which gave rise to even richer discussions. Where moderate management was applied to the questions asked, the management of the discussions themselves was very limited, in order to allow the participants to reflect on the topic in question as naturally as possible and to reduce potential interviewer effect. This low form of managing the discussions was a deliberate choice to get participants to interact with each other rather than with the facilitator. In the study of Bloor et al [46], this finding is supported by Murray [47], one of the pioneers within online focus groups, who found that too high level of interactions from the facilitator can lead to participants answering the facilitator rather than stimulate discussions among participants [46].

### Participant Interactions

In traditional face-to-face focus groups, various techniques can be used to support group processes, promote participant interaction, and focus the discussion of the topic (eg, so-called prompts, which are actions that help to make the conversation flow; in the form of affirmative body language such as nod, mimic, and encouraging expressions or probes that encourage participants to elaborate on their answers such as “And what happened then?” or “Tell me more about this”) [48]. In the online focus groups, where the use of body language was excluded, we adapted traditional interviewing techniques to the web-based universe by using Facebook's *reaction symbols* [31] to nonverbally acknowledge a comment. As in traditional focus groups, online focus groups may also face challenges with some participants retaining and not interacting naturally with the other participants. This was also the case in this study, where some participants or groups did not participate as actively as others who were very active in the discussions and interacted mutually with each other. Surprisingly, in this study, the youngest group (age group of 18-25 years) had remarkedly less interactions than the other groups. A cautious suggestion can be that this age group felt it harder to relate to the topic of timing of motherhood, as they are not at a time in their lives where motherhood seems as relevant yet, as it can be in the other age groups. However, more research is needed to explore how younger women reflect upon motherhood.

In this study, we discovered different behavioral patterns among the participants. The majority of the participants responded to all the questions uploaded by the facilitator and interacted vividly with one another by using text, photos, videos, reaction symbols, and emojis to comment on each other’s posts. Often, an individual opinion was developed through a discussion with other participants, which in a constructivist perspective can be seen as data are being socially constructed among participants and framed during the interpersonal exchange of opinions and different perspectives [45]. In addition, some participants made updates on other thoughts of motherhood at their own initiatives, which emphasizes that there was a great deal of interest in discussing the topic of motherhood while providing good group dynamics and demanding discussions within the group, without getting too far off the original topic. Clemensen et al [49] define a participant who starts a communication thread as an updater, and in this study, we identified a couple of updaters within each age group, who helped stimulating group discussions and fostered interaction with less active participants. In contrast, we also identified a few passive participants, whom according to Bloor et al [46] could be considered as so-called lurkers who only contributed with a presentation of themselves on the first day and did not contribute to the discussions at all. Remarkedly, they read all the posts uploaded by the facilitator, were online daily, responded to the debriefing questionnaire, and did not withdraw from the study. This indicates that even though passive participants do not necessarily interact or contribute with comments, they can still have a sense of participation, which shows how participation can take place at different levels. Although Bloor et al [46] implied that it is easier for the researcher to encourage participation from those who read, but not respond, when doing asynchronous online focus groups, we found it difficult to point out the low responders, as it was hard to figure out whether the low responders were simply not online at the same time as other participants or whether they did not want to comment. It is the facilitator’s role to try to involve these lesser active participants, but in this study, we found it problematic to address questions directly to a specific participant without the participant feeling designated, with the risk that some participants were hiding and just reading the comments of others without participating in the discussions themselves.

### Use of Emojis and Reaction Symbols

In the written language, there is a lack of nonverbal expressions such as facial expressions, body language, and emphasis on words. When participants communicated with each other in the online focus groups, emojis or reaction symbols were used several times, typically when participants needed to denote a feeling, express humor or irony, or to emphasize a point. The use of emojis and reaction symbols are examples of how verbal communication in a face-to-face focus group can be translated into web-based language, showing how it is possible to express nonverbal communication without the physical presence. Since all participants in this study were familiar with Facebook, the use of emojis and reaction symbols appeared natural and did not cause obvious misunderstandings between the participants. The use of emojis, reaction symbols, and punctuation was incorporated in transcripts and contributed to richness in data and gave a greater understanding for group dynamics, for example, the use of abbreviations (eg, OMG for Oh my God) to express surprise or dismay, or punctuation (eg, ‘!!!’), when something needed to be emphasized.

When using reaction symbols (eg, interacting with a heart), it seemed as if participants were emotionally closer than what online media usually foster. For example, when Catherine discloses her family history with mental problems, which causes her to consider whether she should become a mother or not herself, other participants responded to her by conveying care online with the use of reaction symbols. This indicates that web-based communication can elicit a different range of feelings, for example, receiving care from other online participants. This finding is supported in the study of Bloor et al [46] by Stone (1995) who argues that technology can convey more than just words, in the sense of smell, touch, sight, etc, and that interactions in cyberspace are social in character.

### Reflections on Future Motherhood

More than half of the women stated that participating in online focus groups made them feel as a part of a community. This indicates that there is a possibility that women of reproductive age have unmet needs for discussing thoughts on future motherhood, including timing of motherhood, before they decide whether and when to start a family. Online communities have the potential to give women of reproductive age a feeling of belonging and a voice in the debate of motherhood. Prefertility web-based forums can be a solution to address these needs, but further research needs to be done to explore this. Two participants stated that they spent more time than estimated to engage in the discussions. When studying a sensitive topic as the timing of motherhood, it can be necessary to allow more time for reflection than the estimated 15-20 minutes suggested in this study. Lack of time can explain why some participants did not respond as much as others.

### Strengths and Limitations

This study has several strengths, including the insight in a rather unexplored field discussing the timing of motherhood among a group of women of reproductive age who had not yet had children—a topic that many people can consider as personal and sensitive. Another strength is the innovative approach of this study using online focus groups to collect qualitative data on social media with women of reproductive age. Facebook was found to be a suitable platform for collecting qualitative health data, as it was easy for recruitment, cost-effective, well-known, and easy to use by participants and researchers. However, despite these strengths, there are some limitations that need to be considered. A limitation of this study was that we only included women who had a Facebook profile, which meant having access to internet was a prerequisite for participation. In Denmark, where 98% of the population has internet access in their homes, access to participation was not a challenge, but in other settings, internet access should be considered. Another limitation addresses the absence of complete anonymization. When participants used their private Facebook profile, they, to some extent, accepted not being anonymous to other participants, depending on the degree to which they had limited their privacy settings on Facebook. This differs from other types of web-based research, where you typically guarantee participant anonymization, which is not possible to the same extent when doing research on Facebook [50] and why confidentiality in the processing of data is essential, especially in vulnerable or stigmatized groups. Given the reservations about not being anonymous, Lijadi and van Schalkwyk [34] describe that the advantage of participants using their own identity (being nonymous) allows the researcher to verify the participant’s authenticity and member identity. A concern associated with conducting online focus groups on Facebook is that research data were subject to Facebook’s privacy policy [51], in which Facebook claims to collect data on communication on the platform, leaving the researcher with less control over the data. All participants had accepted these terms as they were Facebook users prior to participation in this study. As the majority of participants had a high educational level (medium higher or higher education), women with lower education were not represented to the same degree in this study. However, where we often see a connection between less educated people and the development of various diseases, we see the opposite in the reproductive context, based on the fact that it is often highly educated women who have children at advanced maternal age. Conducting online focus groups on Facebook proved to be particularly beneficial during the COVID-19 pandemic, where data collection was compromised in relation to arranging physical meetings. Thus, web-based research on social media makes it possible to connect with participants even under special circumstances. By showing the advantages as well as the disadvantages of conducting online focus groups in a social media setting, we want to stress that online focus groups is not a replacement for traditional focus groups but shall be considered as an independent alternative method for researchers to use when the topic of interest is suitable for web-based research.

### Conclusion

The results of our study show that Facebook is an eligible platform to access qualitative data from women of reproductive age, as we succeeded in recruiting women for this study and collecting qualitative data. Conducting online focus groups on Facebook is an eligible method to access qualitative data from women of reproductive age within health research, especially when participants have access to the internet and are familiar with the platform. Overall, participants were positive toward being a part of an online focus group, and the majority of the participants considered it an advantage to meet on Facebook instead of a physical meeting. Online focus groups have the potential to give women of reproductive age a voice in the debate of motherhood. Further research must be done to explore the impact of conducting qualitative health research on web-based platforms.
